# 3Mo: A Model for Music-Based Biofeedback

**DOI:** 10.3389/fnins.2016.00548

**Published:** 2016-12-02

**Authors:** Pieter-Jan Maes, Jeska Buhmann, Marc Leman

**Affiliations:** Department of Art, Music and Theatre Sciences, Institute for Psychoacoustics and Electronic Music, Ghent UniversityGhent, Belgium

**Keywords:** sonification, auditory biofeedback, music interaction, reinforcement learning, predictive processing

## Abstract

In the domain of sports and motor rehabilitation, it is of major importance to regulate and control physiological processes and physical motion in most optimal ways. For that purpose, real-time auditory feedback of physiological and physical information based on sound signals, often termed “sonification,” has been proven particularly useful. However, the use of music in biofeedback systems has been much less explored. In the current article, we assert that the use of music, and musical principles, can have a major added value, on top of mere sound signals, to the benefit of psychological and physical optimization of sports and motor rehabilitation tasks. In this article, we present the 3Mo model to describe three main functions of music that contribute to these benefits. These functions relate the power of music to Motivate, and to Monitor and Modify physiological and physical processes. The model brings together concepts and theories related to human sensorimotor interaction with music, and specifies the underlying psychological and physiological principles. This 3Mo model is intended to provide a conceptual framework that guides future research on musical biofeedback systems in the domain of sports and motor rehabilitation.

## Introduction

In this article, we consider the potential of music as feedback system to support and influence the psychological and physical demands inherent to sports and motor rehabilitation tasks. Musical biofeedback may be considered a particular case of auditory biofeedback, or “sonification,” Sonification is commonly defined as the transfer of data, and data relationships, into non-speech audio for the purpose of communication and interpretation (Kramer et al., 1999; Hermann et al., 2011). Starting in the 1970s (Zaichkowsky, 1982), the use of sonification, or auditory feedback, in the domain of sports and motor rehabilitation has substantially progressed over the past 20 years (Huang et al., 2006; Dubus and Bresin, 2013; Giggins et al., 2013; Sigrist et al., 2013; Kos et al., 2015). Typically, sonification is used to enhance self-awareness of physiological processes and physical motion in order to regulate and control these in most optimal ways. Thereby, auditory biofeedback systems commonly map physiological and physical quantities to psychoacoustic (sound) parameters, such as loudness, pitch, timbre, and rhythm (Hermann and Hunt, 2005; Dubus and Bresin, 2013). Studies that explicitly use music as auditory biofeedback however are relatively scarce (Bergstrom et al., 2014; Moens et al., 2014; Van Dyck et al., 2015).

**Figure 1:**
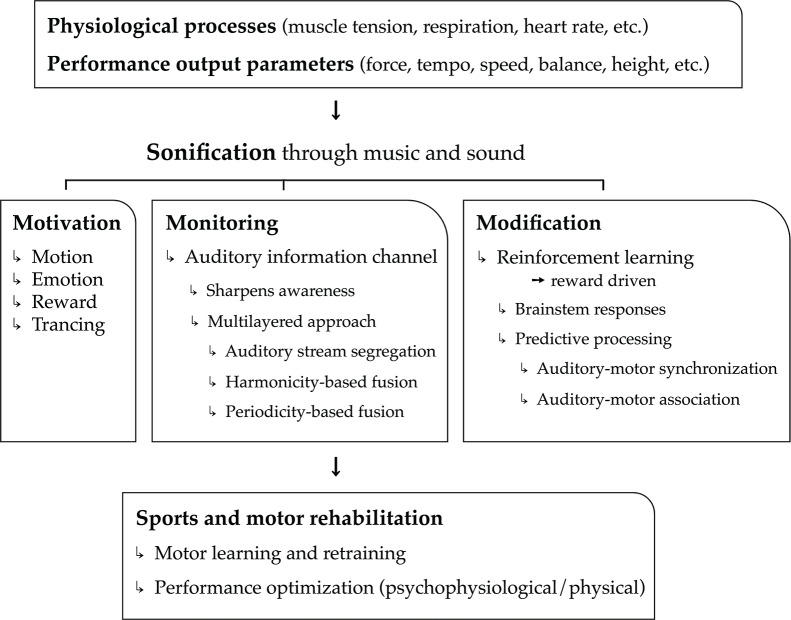
**Schematical overview of the 3Mo model (MOtivation, MOnitoring, MOdification) delineated in the article**.

In the current article, we put forward the hypothesis that music is highly convenient as real-time feedback of physiological processes (cardiovascular, respiratory, electro-dermal, etc.), motor kinematic and kinetic processes, and performance parameter output (speed, force, height, etc.). Used as biofeedback system, we assert that music can have a major added value, on top of mere sound, to the benefit of psychological and physical support and optimization of sports and motor rehabilitation tasks. The sports and motor rehabilitation tasks relate mainly to motor exercise, learning, relearning, and actual performance (including warming-up and cooling down). Argumentation in support of our hypothesis is structured according to three main functions of music and musical biofeedback: the power of music to motivate physical activity (i.e., motivation), the ability of musical biofeedback to monitor physiological and motor processes (i.e., monitoring), and the potential to use music to modify (i.e., optimize) these processes (i.e., modification). These three core functions—motivation, monitoring, and modification—outline the three pillars of our model, hence the name “3Mo model” of which a schematic overview is presented in Figure 1.

The first function concerns the “power” of music to motivate people; music stimulates people to get physically active, it induces emotions and moods, and it modulates attention and feelings of pain and exertion through “deep listening” (“trancing”). A second function is a more common one in the field of sonification research and relates to monitoring. The real-time monitoring of physiological processes, motor processes, and performance output parameters may sharpen self-awareness and drive self-regulation. In this section, we demonstrate that music is particularly relevant for providing real-time biofeedback of multiple, concurrent (i.e., multilayered) physiological and physical processes. A third function pertains to the possibility of music to reliably modify physiological processes and motor behavior toward specific goals through reinforcement learning. This function relies on principles related to brainstem responses and sensorimotor predictive processing.

In support of each function, we collect concepts, theories, and empirical evidence. In that respect, the article introduces a novel model that brings together already existing theories. On top of that, some novel ideas are included that have received only limited evidence so far in the context of musical sonification for sports and motor rehabilitation purposes. This relates to ideas of multilayered musical biofeedback (see Monitoring by Musical Biofeedback: “Monitoring”) and the use of prediction and musical reward principles to endow motivational qualities (see Motivation by Musical Biofeedback: “Motivation”) and modify physiological and motor processes (see Modification by Musical Biofeedback: “Modification”).

Our approach is based on the idea that interaction with music is empowering (Leman, 2016). It gives music a central role in the development of expressive interactive machines that work with biofeedback. The concepts and theories used have strong links with musicological research, touching upon music performance, music emotions, music analysis, and even ethnomusicology. However, they are rooted in evidence coming from broader academic disciplines—including cognitive science, (neuro)physiology, and motor control—to provide an explanation of underlying psychological and physiological principles. These concepts and theories are brought together into a general model to make a strong case for the role of music in biofeedback systems. This model is intended to provide a conceptual framework that guides future research and practice. However, the model is still preliminary and needs further testing to proof its validity.

## Motivation by musical biofeedback

In the context of sports and motor rehabilitation, situations of high endurance, pain, fatigue, and “repetition-to-boredom” are ubiquitous. Motivation—or, the *will* to act—is therefore an indispensable aspect to persevere in these situations. We argue that the use of music and sounds coupled to motor behaviors—i.e., sonification—is particularly powerful as it may take advantage of the strong motivational qualities inherent to people's interactions with music. In the following, we show how music may stimulate active behavior, elicit strong emotions, elicit feelings of reward, and induce altered states of consciousness (trance), contributing to motivation.

### Music and motion

A prominent characteristic of music is that it motivates people to get physically active. Almost everyone has experienced the compelling drive to tap the feet, nod the head, sway arms and hips, or dance along with music. Gesturing along with music is very common and bodily renderings of musical and sonic features may be quite elaborate, encompassing musical beat, melody, dynamics, phrasing, etc. In this paragraph, we outline two neurophysiological mechanisms that contribute to these phenomena. A first one is an *arousal* mechanism within the central nervous system, a second one a *motor resonance* mechanism.

Arousal has been attributed to the functioning of the brainstem, more in particular the reticular formation (Pfaff, 2006; Juslin and Västfjäll, 2008; Pfaff et al., 2012). It is demonstrated that increased arousal occurs in response to salient, unexpected sensory events and promote increased sensory alertness, emotional reactivity, and instinctive or learned motor activity.

On the other hand, bodily renderings of musical and sonic features may rely on a motor resonance mechanism. Apart from overt movement responses to music, there is ample neurophysiological evidence that passive listening to music automatically co-activates motor regions within the brain (for examples, see Maes et al., 2014). A particular interesting musical feature shown to elicit body movements and motor cortex excitability in listeners is musical groove (Janata et al., 2012). These phenomena are considered motor resonance or ideomotor effects (related to theories on mirror neurons), referring to the process whereby perceptual (here, auditory) events trigger automatic muscular reactions based on previously established action-perception relationships (for more details, we refer to Maes et al., 2014). In addition to this effect of becoming physically active, motor resonance may create the illusion of having control over the actual skillful production of music and sounds (cf. sense of agency).

Although arousal and motor resonance mechanisms are presented here as different mechanisms, they link in that they rely both on prediction processes in the brain. We argue that it is exactly because of the dynamics of predictability and surprise (and, tension and release) inherent to most music that arousal and motor resonance mechanisms are “set into action,” become relevant in explaining music-induced body movement, and eventually can be deployed for sports and motor rehabilitation purposes.

Other than affecting the timing of people's movements, music has also been shown to affect the amount of vigor in people's movements. A walking experiment, where people were instructed to synchronize their steps to the beat of songs at 130 BPM, revealed a significant effect of the type of music on step size and thus on walking velocity (Leman et al., 2013). It seemed that even though all songs were at the same tempo, some music had a relaxing effect, decreasing the step size, and other music had an activating effect, actually increasing the step size compared to the average step size of walking to a metronome.

This effect of music was also found in a self-paced walking experiment were people were not instructed to synchronize with tempo-matched music (Buhmann et al., 2016). Analysis of the musical features attributing to the velocity effect of the music, showed that music with a recurring pattern every four beats had an activating effect on kinematic responses, resulting in bigger stride lengths compared to walking without music. On the other hand, music with recurring patterns every three or six beats, had a relaxing effect on the kinematic responses, resulting in smaller stride lengths compared to walking without music. Such emphasis on ternary aspects of the meter could either be due to a 3/4 meter in songs, or it could be the result of syncopating melodies. In both cases this ternary recurring pattern in the music seems to counteract the regular flow of a binary walking pattern. Results indicate, that although musical groove elicits body movements, these movements may not always contribute to the forward movement in walking or running. The expressiveness in high-groove music is often represented by non-binary recurring patterns in the rhythm or melody of the music. In order to boost performance in cyclic, binary movements, such as walking or running, caution is needed to select the most suitable kind of music.

Another outcome of the study by Buhmann et al. (2016) emphasizes the motivational aspects of music-movement interaction. Subjects were asked to rate different motivational aspects of all the songs they heard during their walk. This was done with a BMRI-2 test (Karageorghis et al., 2006). Songs that increased walking velocity were rated significantly higher with respect to motivation, thus revealing a close link between the effect of music on walking velocity and the power of music to motivate.

### Music and emotion

Next to motion stimulation, music is well-known for eliciting emotions. More even, emotional expression and regulation is often heard as prime motivation for people's engagement with music. There is ample research evidence showing that listening to music can have an effect on activity in the limbic system, considered to be the brain's emotional core (Blood and Zatorre, 2001; Koelsch, 2010; Peretz et al., 2013), and might lead to that “spreading gooseflesh, hair-on-end feeling” better known as “chills” (Panksepp, 1995). Used as a biofeedback system, it has been shown that music may modulate physiological arousal (Bergstrom et al., 2014). It is important to acknowledge that emotional responses to music and sound are tied to individuals' personal traits, preferences familiarity, and music-related autobiographic memories (Kreutz et al., 2007; Barrett et al., 2010). Therefore, it is recommended to take these aspects into account in sonification designs for sports and motor rehabilitation. However, research also delineated surface features in music and sound, such as consonance, tempo, mode, and texture, that affect musical responses more universally, or at least cultural-wide (Webster and Weir, 2005; Fritz et al., 2009; Weninger et al., 2013). An interesting phenomenon is the unpleasant sounding of dissonance, in contrast to consonance. Low-level, sensory dissonance is based on the sensation of “beating” and “roughness” of interacting partials of (musical) sounds. Although it is still an ongoing debate whether a dislike of dissonance is a truly innate or rather learned phenomenon, Juslin and Västfjäll (2008) attribute this phenomenon along with our responses to other basic acoustic qualities, such as fast, loud, noisy, and very low- or high-frequenced sound—to brainstem reflexes. These researchers consider these reflexes innate and automatic causing emotions, which may further act as positive or negative reinforcement of behavior. This aspect of automatic behavioral reinforcement mediated by emotional responses is highly relevant for sonification purposes in the context of sports and rehabilitation (see Modification by Musical Biofeedback). As motor behavior is attracted toward pleasant sounding qualities, one may link these qualities to desired motor behavior (positive reinforcement), while unwanted motor behavior is then matched to unpleasant sounding qualities (negative reinforcement).

### Reward

In the context of sports performance and motor rehabilitation, people need to be motivated to learn in order to develop new behaviors and solve problems in order to reach specific goals. In this context, the just-mentioned concept of reinforcement in relation to music and musical emotion may be highly relevant. The aspect of reinforcement in relation to learning of new motor skills is closely tied to the concept of “reward.” In reinforcement learning, people are not told exactly what to, but it is assumed that people will act and behave so as to maximize their received reward. In other words, reward is considered a prime motivator, “reinforce,” or “attractor” of actions and motor behavior by inducing feelings of pleasure and happiness. Hence, learning environments are setup in a way that wanted behavior yields maximal reward and people are stimulated to discover this by themselves. Reward and punishment are thus considered constraints guiding motor behavior toward specific goals. This principle of reinforcement learning will make out the core of the third component of our model, namely motor modification. In the corresponding section, we will go into more detail on the physiological principles of reward, and on the musical features and principles that can exert a strong rewarding force in people's engagement with music.

### Trancing

Complementary to emotions and mood induced by music listening are aspects relating to absorption, dissociation, and trance (Becker, 2004; Herbert, 2011; Schäfer et al., 2013; Clarke, 2014). These aspects point to changes of awareness and consciousness that occur when people are “deeply” engaged with music, either by listening or performing. In a seminal work by Herbert (2011), the concepts of absorption and dissociation are thoroughly defined. They are determined subjective qualities subsumed within the experience of altered consciousness, or trance experience. Absorption refers to the experience of being completely occupied with the musical stimulus, without requiring any mental effort. Dissociation involves mentally cutting off from surroundings and extraneous thoughts. The benefits of using these subjective qualities in sonification designs in the context of sports and motor rehabilitation are ample. Being detached from internal and external “concerns” may lead to a calming, pleasant, and effortless experience, in which a focus could be maintained on the physical or mental task at hand. At the same time, as Herbert emphasizes, trance experience helps focusing on music that, via both emotional and formal qualities, may interact with the physical or mental task at hand. More specifically, Becker refers to DeNora's (2000) concept of music (or read, sonification) as a prosthetic technology of the body that provides organizing properties to which a range of bodily and mental processes can be entrained. Also, highly relevant in the context of sports and rehabilitation, is the link made between trance experience and the insensitivity to pain and fatigue, and high physical endurance. Becker (2007) provided an explanation of altered pain responses in trance experiences based on theories of the biology and neurophysiology of consciousness (e.g., Damasio, 1999). According to Damasio, the term “emotion” designates a specific autonomic physiological response—i.e., not under conscious control. Becker (2007) believes that persons engaged in a trance experience take voluntarily control over the physiology of emotional arousal, leading to a reduction of normal pain response and fatigue, and high physical endurance.

In the field of sports, a range of studies on music and locomotion has revealed positive effects of music in general, and synchronous music in particular, similar to the effects of a trance experience. With respect to psychophysical outcomes, significant increases in time-to-exhaustion (TTE) were uncovered when running to both motivational and neutral music, compared to running without music (Terry et al., 2012). In addition, significant effects of music on ratings of perceived exertion (RPE) were found when running at sub-maximal intensities (Bood et al., 2013). Typically, there are two common explanations given to account for these findings. A first one is based on an informational processing model claiming that proprioception information (effort sense) and affective information (emotional quality of music) is preprocessed in parallel (Rejeski, 1985; Boutcher and Trenske, 1990). As only a certain portion of information can be processed at once, music may distract from focusing attention on the internal sense of effort in vigorous activities. A second explanation is based on the concept of agency. As shown by Fritz et al. (2013), musical agency, defined as the performance of bodily movement with the intention to modulate expressive features of the musical feedback (timbre, loudness), may have effects on perceived exertion during a physically strenuous task.

Concerning physiological outcomes, lower oxygen consumption has been reported for athletes running with music compared to running without acoustic stimuli (Terry et al., 2012). The use of music is associated with better running economy although more evidence is needed to support this claim. Another effect on physiology has been revealed on heart rate: treadmill running with music at (near-) maximal RPE resulted in increased heart rates (Bood et al., 2013). This implies that music helps runners to perform at higher intensities. Furthermore, a study on the psychophysiological effects of synchronous vs. asynchronous music during cycling reported positive effects of synchronous music over asynchronous music (Lim et al., 2014). Although synchronizing movement to rhythmic stimuli did not reduce metabolic cost, it did lower limb discomfort and had a stronger effect on arousal than compared to asynchronous music.

## Monitoring by musical biofeedback

According to its definition, sonification pertains to “the use of non-speech audio to convey information,” whereby data relations are transformed into “perceived relations in an acoustical signal for the purpose of facilitating communication or interpretation” (Kramer et al., 1999). In the present article, we approach sonification in relation to physical activities in the domain of sports and rehabilitation. In this context, one often speaks about auditory biofeedback instead of sonification. Thereby, sound and music is considered an auditory information channel that may—through real-time feedback—sharpen the awareness of physiological processes (cardiovascular, respiratory, electro-dermal, etc.), motor kinetic and kinematic processes, performance parameter output (speed, force, height, etc.), and their relationships. New technologies enable measuring these processes and parameters, and translating them into auditory information streams. Accordingly, attention might be directed to previously non-conscious operations of the body (“body schema”) and it becomes possible to affect these operations (Metzinger, 2003). This is particularly interesting, as physiological processes, motor processes, and performance output parameters tend to efface itself from conscious experience in most behavior. Augmenting the natural monitoring mechanism with technologies that sonify these processes and parameters may assist self-regulation leading to optimal performance quality and efficiency in the domain of sports and rehabilitation.

An early experiment, illustrating the potential of sound to make people aware of muscle activation was conducted by Basmajian (1963). Slight contractions of skeletal muscles often recruit only a few motor units that may not be apparent through normal “proprioceptive” feedback. In Basmajian's experiment, gentle contractions of a hand muscle (*abductor pollicis brevis*) were made apparent to the subjects through combined auditory-visual feedback. Basmajin found that, over time, subjects obtained fine voluntary control of individual motor units, so as to be able to perform various “tricks,” such as the production of rhythms, doublets, and roll effects. Interestingly, aural feedback proved more useful than visual feedback in learning and retaining these skills. In sports, biofeedback was introduced in the 1970s for the purpose of stress regulation, motor retraining, and motor performance optimization (Zaichkowsky, 1982). In these early experiments, measurement tools were used to monitor a wide range of physiological functions (e.g., heart rate, blood pressure, and muscle tension) in order for subjects to learn to voluntarily control these functions most optimally, leading to lower anxiety levels and/or improved motor performance. Most of the auditory biofeedback systems that have been used in previous research and practice are limited to providing auditory feedback of one specific process or parameter. However, in the following, we pinpoint the relevance in sports and motor rehabilitation to be able to coordinate multiple biological and/or motor processes simultaneously. We argue that the use of sound and music is particularly suited to assist in this task, through multilayered auditory feedback strategies.

### Multilayered musical and auditory biofeedback strategies

Basically, music hits our eardrum as a highly complex mixture of air vibrations coming from different sound sources and sound layers, and affected by the acoustic environment (e.g., reflections). In the following, we describe into more detail how the human auditory processing system deals with this complex mixture of sounds and consequently, how sonification strategies may capitalize on these principles for the benefits of sports and rehabilitation research and practice.

#### Auditory stream segregation and integration

Auditory stream segregation and integration concerns the fusion and fission of sound streams. The human auditory apparatus is particularly well accustomed to decompose a complex mixture of sounds into separate units (cf. auditory perceptual objects), through a psychoacoustic process called “auditory stream segregation,” “fission,” or “auditory scene analysis” (Bregman, 1990). Auditory streams thereby appear as emergent patterns: they result both from the acoustical configuration from sound and from the auditory-brain disposition to process those patterns and generate percepts. In speech perception, this process relates to the “cocktail party problem,” denoting human's ability to focus on a single voice within an auditory noisy environment (McDermott, 2009). In the context of music, auditory streaming segregation enables for instance to discern different instrumental sections in a symphonic work, a rock song, or electronic dance music. Earlier experimental research has pinpointed specific physical properties that support the separation of music into different distinct sources, such as pitch, timbre, loudness, tempo, and rhythm (Miller and Heise, 1950; van Noorden, 1975; McAdams and Bregman, 1979). A simple example is a sequence (ABABAB) of subsequent low (A) and high (B) pitches, which segregates as separate streams of A A A and B B B depending on duration and pitch interval. New insights into auditory anatomy and physiological processes have deepened our understanding of auditory stream segregation (Snyder and Alain, 2007; Bizley and Cohen, 2013), with an important role attributed to predictive information processing (Grossberg, 1999; Winkler and Schröger, 2015).

#### Pitch-harmony-tonality-based emergence

Pitch, chord harmony and tonality are based on the fusion of acoustical harmonic components. Consequently, they can be considered as outcomes of an emergent process based on acoustical configurations and auditory disposition. The main effect is that different auditory perceptual objects may blend together into a single auditory perceptual object; cf. harmonicity-based fusion principle (McAdams et al., 2004). Exemplary for this principle are the tones produced by musical instruments. Typical for vibrating strings (cf. string instruments) and air columns (cf. wind and brass instruments) is that they create multiple, harmonically related frequency components (partials) that combine together into complex tones. More in particular, such components contribute to the perception of *pitch* (based on the lowest harmonic fundamental if present, or based on the harmonic residue if absent)—and a series of overtone frequencies or harmonics whose amplitudes determine perceptual *timbre*. Another example is a major chord, which is composed of three instrumental tones (e.g., piano, guitar, etc.) being a root tone (e.g., C4), with on top a major third (E4), and perfect fifth (G4). These tones fuse because the harmonic patterns of these tones fuse into a harmonic residue that supports a bass note (C2) as root of the chord.

#### Rhythm-periodicity-based emergence

Rhythm, or periodicity in general, has similar emergent characteristics as harmonicity, but they occur in the temporal domain (cf. Stockhausen, 1957/1959). Music contains overlapping, periodically repeated acoustic-perceptible patterns, such as beats, measures, and phrases that define overall hierarchical temporal structures. Typically, the periodicities of these patterns are related, in the sense that they are proportional to integer multiples (Butler, 2006). Pulses or beats, groups of two or three beats that define a measure, and even sequences of irregular meter (containing beats in successive patterns of 2, 3, and 3 beats) are quite common in music. What emerges at this temporal level is again based on the acoustical configuration and the auditory-brain disposition. Making an abstraction of the actual musical content, it is possible to consider the manifold of periodic acoustic-perceptible patterns as a series of phase-locked oscillators, with an integer relationship of frequency. Although these different layers may be distinguished in terms of source and timbre, their temporal integration contributes to the perceptual grouping and fusion of the stream of auditory events.

Research demonstrated that musical patterns characterized by integer-related temporal periods (i.e., metrical layers) are manifested in spontaneous movement or dance to music (Naveda and Leman, 2009, 2010; Leman and Naveda, 2010; Toiviainen et al., 2010). In their studies, Naveda and Leman investigated the relationship between Samba music and dance. Using the method of Periodicity Transforms (PT), they decomposed Samba dance movement into its constituting periodicities, matching the metrical layers within the music. Samba movements were found to have their energy level mainly concentrated within 2-beat periods. Based on these “fundamental” periods, repetitive spatial movement trajectories (i.e., “basic gestures”) could be extracted for each body part. Based on this, the authors proposed a sonification of the resultant basic gestures, which led to an auditory reproduction of traditional rhythmic structures of Samba music (Naveda and Leman, 2008).

#### Relevance for sonification purposes

Based on the above-described emergent principles that are typical for music—respectively, auditory stream segregation, Pitch-Harmony-Tonality-based emergence, and Rhythm-Periodicity-based emergence—we outline three strategies for the purpose of multilayer sonification of physiological and/or motor processes.

A first strategy is based on the human ability to process different auditory layers on top of each other. By assigning different layers of auditory feedback simultaneously to different physiological processes, motor processes, and performance parameters, one may achieve to raise awareness of how these processes work together in relation to performed output and feeling. Coordinated behavior can thus be sonified by means of different interacting layers (Naveda and Leman, 2008).

Muscle synergy, or ensemble muscular activation, describes the complex integration of muscle groups and nerves that underlie the most common motor behaviors. Current research gained insights into how muscle synergies are encoded into the nervous system (Bizzi and Cheung, 2013). The topic of muscle synergy is of major importance for sports and motor rehabilitation (Barroso et al., 2015; Ting et al., 2015). Imbalances in multi-muscle coordination may lead to impaired performance and even injuries. We claim here that the “orchestration” of muscle synergies may be realized through a coupled, well-balanced auditory “symphony” (i.e., multilayer auditory biofeedback). Sonification may enhance awareness of the relationship between the different muscles, and may hint for changes in motor behavior to perform optimally and avoid muscle congestion and injury.

This first strategy focuses on making different physiological/motor processes and performance parameters explicit in corresponding auditory biofeedback streams, thereby relying on human auditory stream segregation skills.

In a second strategy, we rely on before-mentioned fusion effects, in which different auditory layers blend together into a single auditory perceptual object. Here the focus is on the “end-product” (i.e., the outcome) of processes working together, instead of on the explicit contribution of each process.

An example of a pleasant sounding outcome is the combination of three pure tones that are harmonically related, e.g., 440, 880, and 1320 Hz. Another example is a musical major chord composed of a root note, major third, and perfect fifth. In the fourth section, we go into more details about musical and psychoacoustic principles that relate to reward. This approach is representative for the reinforcement learning method mentioned before. The idea is that optimal coordination of processes lead to a pleasing auditory outcome (cf. reward), while less optimal coordination of processes lead to gradual degrading of this pleasing auditory outcome (cf. “punishment”). This idea may also be applied to the sonification of the performance outcome of different persons, for example when they are engaged in a synchronized activity. The degree of synchronization may be rewarded by sonification (Varni et al., 2010; Demey et al., 2013).

A third strategy relates specifically to temporal periodicity-based fusion. We mentioned earlier that music often contains repeated patterns of which periodicities are proportional to integer multiples. Hence, we proposed to represent musical content as a series of phase-locked oscillators. This idea of phase-locked oscillators is interesting in light of the various physiological and motor processes engaged in sports practice. Many physiological processes (such as breathing and heart beat) and motor processes in sports are characterized by periodic patterns, and may well be considered as oscillatory systems and biological oscillators. Interestingly, research indicates that some of these biological oscillators tend to synchronize to each other at different period proportions; e.g., cardiovascular-respiratory (Schäfer et al., 1999), cardiovascular-motor (Niizeki and Saitoh, 2014), and respiratory-motor (McConnell, 2011). McConnell (2011) pinpointed the importance of coordinating breathing and motor rhythms to maximize comfort and (metabolism) efficiency in running, cycling, and rowing performances (Hoffmann et al., 2012). The fact that both music and biological processes within an individual may be conceived as a multilayered oscillatory system is interesting. It suggests that music may assist in simultaneously synchronizing multiple biological oscillators at different periods. Note however that the proportions of the periodicities of biological oscillators change depending on exercise intensities. For instance, The breathing-pedal cadence ratio of competitive cyclists shifts from 1:3 to 1:2 as exercise intensity or duration increases (McConnell, 2011). This entails that, for synchronization purposes, proportions of acoustic-perceptible periodicities within the music should change accordingly in order to match corresponding biological periodicities.

Although different periodicity layers in a song might be used to sonify different physiological and motor processes in movement, these periodicity layers might all somehow be related (integer multiples) and impact each other. Depending on the sort of relation, they either enhance one another or not. Evidence for this effect was given in the studies by Leman et al. (2013) and Buhmann et al. (2016), where musical layers with certain multiples of the beat tempo had an increasing effect on velocity (e.g., a periodicity of once every four beats), whereas other multiples had a decreasing effect on velocity (e.g., periodicities of once every three or six beats). In regard of using music to manipulate specific physiological or motor processes, we therefore need to consider that different multi-layer tempi can enhance or diminish the amount of strength a subject applies in the movement or breathing that is being monitored by the music.

## Modification by musical biofeedback

The monitoring of ongoing motor behavior by means of music and sound information refers merely to the direct transfer of behavioral data (basically physiological, movement, and performance output data) into auditory form for the purpose of increasing self-awareness of one's own behavior. However, monitoring may often become an assistive component of biofeedback systems that target the modification of motor behavior. Basically, two approaches can be distinguished here.

The first approach requires that the learner has an explicit representation of the target behavior (i.e., goal) to which ongoing behavior can be compared. Both the target behavior and the actual, ongoing behavior can be represented through music or sound patterns to allow such comparison. Learning and adaptation then consists in reducing the error between ongoing and target behavior; a form of goal-driven learning (Ram and Leake, 1995). As Ram and Leake (1995, p.1) point out, the process of goal-driven learning is guided by reasoning, in order to make good decisions about when and what to learn, to select appropriate strategies for achieving the desired learning, and to guide the application of the chosen strategies. In this regard, monitoring—i.e., sharpening self-awareness—becomes an essential precondition for people to modify their behavior. And therefore, the process of modification cannot be disconnected from the ability to monitor oneself. This is the typical approach in motor learning and rehabilitation research.

In the following, we propose and focus on an alternative approach to behavior modification that does not rely on self-monitoring; in other words it does not require that the learner has an explicit representation neither of the own behavior nor of the target behavior. In that regard, modification becomes disconnected from a direct transfer of behavior parameters (physiology, movement, and performance output) into audible form. Instead of relying on such explicit representations, learning and adaptation is reward-based, using reinforcement principles. In reinforcement learning, people are not told exactly what to do (i.e., the goal), but it is assumed that people will act and behave so as to maximize their received reward. The human reward system is understood as a collection of neural structures that contain dopamine-secreting neurons in the midbrain with pathways to other brain structures, such as the striatum, the hippocampus, and the prefrontal cortex (Schultz, 1999; Wise, 2004). Music is a particularly relevant phenomenon in this context as it is a potent source of pleasure and reward for most people (Dubé and Le Bel, 2003). Previous studies have demonstrated that music listening can activate the human reward system (Blood and Zatorre, 2001; Menon and Levitin, 2005; Alluri et al., 2015). In line with the core idea of reinforcement learning, we argue that pleasant and rewarding states promoted by music may function as an attractive force (“attractor”) of motor behavior. In the following, we delineate two principles regulating musical reward that can be exploited in strategies for modification of motor behavior. One is based on auditory brainstem responses (“brainstem-driven reward”), the other on predictive processing (“prediction-driven reward”).

### Brainstem-driven reward

The human brainstem is an evolutionary old part of the nervous system. Automatic brainstem responses occur very early in the brain's processing of auditory information. They are typically responses to auditory events that signal alert to the presence of a potential threat. These auditory events involve sounds that are sudden, loud, dissonant, noisy, very low- or high-frequenced, or feature fast temporal patterns (Juslin and Västfjäll, 2008). Mediated by brainstem activation, these sounds have effects on attention and physiological arousal and eventually, on human behavior (e.g., fight-or-flight response). According to Juslin and Västfjäll (2008), if arousal is too high, listeners will experience the sounds and music as unpleasant and reject them. Hence, listeners will be attracted to music that induces an “optimum” level of physiological arousal. Interestingly, this mechanism may be used as a sonification strategy to influence behavior toward specific goals. Central in this strategy is to associate wanted behavior to pleasant auditory states and processes (cf. reward), while undesired behavior would then prompt unpleasant auditory states and processes (cf. “punishment”). We assume then that motor behavior is spontaneously attracted toward these rewarding (pleasant) states and processes, without explicit knowledge of the target behavior being required (cf. reinforcement learning).

### Predictive processing-driven reward

Within cognitive science, it becomes common to think of the brain as a prediction machine that draws upon learned statistical regularities about the world (Friston and Kiebel, 2009; Clark, 2013). In perceiving, acting, and taking decisions in our daily environment, we are constantly in the process of making predictions of ensuing sensory events, of the probable causes of these sensory events, and of the consequences of actions taken. Theories of music cognition (Huron, 2006) and musical expression (Leman, 2016) draw heavily upon prediction. Importantly, in music research, the ability to anticipate and predict musical events has been acknowledged a potent source of pleasure and reward (Huron, 2006; Gebauer et al., 2012; Zatorre and Salimpoor, 2013). In the following, we consider prediction in relation to auditory-motor synchronization and association learning. We demonstrate, based on empirical evidence, how sonification strategies can be developed based on these principles for the purpose of motor modification.

#### Auditory-motor synchronization

We define synchronization or temporal rhythmic entrainment as the process of adjustment of a motor rhythm (e.g., moving, breathing, etc.) to an external periodic force typically the musical beat. Synchronization to a musical stimulus requires the ability to predict the time at which a musical beat is about to occur. Successful prediction may lead to strong feelings of pleasure and control, such as in dance. Presumably because of that, people often exhibit a spontaneous tendency to synchronize motor rhythms to the musical beat. In that sense, musical beat may function as an attractor of temporal coordinated behavior of humans, such as human locomotion. Sonification through manipulating the timing of the beats can therefore “attract” and influence movement behavior.

In the context of movement rehabilitation, rhythmic auditory-motor entrainment is often referred to as “auditory rhythmic cuing.” There is extensive evidence showing that rhythmic cuing may support timing of upper-limb movement control and gait in persons with motor disorders (Schaefer, 2014; Thaut et al., 2014; Yoo and Kim, 2016). More than temporal control, research points out that rhythmic cuing may lead to adaptation and optimization of spatio-dynamic parameters of motor control, such as smoothing of velocity and acceleration profiles of cyclical movement. Thaut et al. (1999) have provided an explanation of these effects rooted in period synchronization, rather than in phase synchronization. By aligning a repetitive movement pattern to a fixed (and thus, anticipated) rhythmic interval, one obtains accurate and consistent time information throughout the complete movement cycle. In other words, period synchronization allows time to be calibrated against a repeated motor pattern (cf. Maes et al., 2015 discussed further in this article). Correspondingly, the brain may use this time information to optimize spatio-dynamic motor control. Seen from this perspective, beneficial effects of rhythmic cuing becomes evident for the purpose of sports and rehabilitative training (Schaefer, 2014; Thaut et al., 2014).

The fact that musical beat exerts an attractor force on rhythmical motor behavior may also be exploited differently for the purpose of movement monitoring, and eventually movement adaptation and optimization. To exemplify this point, we refer to a music application, called the D-Jogger, that uses sonification of gait tempo for manipulating entrainment (Moens et al., 2014). The D-Jogger system enables a real-time transfer of walking or running cadence data into a corresponding musical tempo. More in particular, the D-Jogger extracts discrete cues in runners' motor behavior—namely, footfalls—to which discrete cues in the music—namely, musical beats—are aligned, both in period and phase. Through continuously adapting the tempo and phase of the music to the footfalls of a runner, D-Jogger monitors the running behavior and creates an audio image of the movement. This monitoring can be done with different alignment strategies that can set a range of coupling strengths between music and movement. In all strategies the initial tempo of a song would be aligned such that it equals the walking or running cadence. In a first strategy, the music is not started in phase. Over the course of the song, music tempo is continuously adjusted to the movement period. In a second strategy, the music does not start in phase as well, but during the song, the music tempo remains stable and the subject entrains to the phase of the song. In a third strategy, the music starts in phase with the movement of the exerciser. During the song, the phase remains fixed, and the subject entrains to the phase of the song, while the music tempo continuously adapts to the period of the movement. In a fourth and last strategy, the music starts in phase and over the course of the song, both music tempo and phase are adapted continuously according to the exerciser's cadence, as such ensuring perfect synchronization between these two rhythms.

Experiments with these different D-Jogger alignment strategies reveal evidence for clear distinctions between different stages of synchronization (Moens et al., 2014). The first stage consists of recognizing the beat and the beat tempo, which has been shown to be the most problematic part of entrainment. The second stage consists of imitating or synchronizing with the beat tempo, a more straightforward component, since a temporal scheme has been established. In the final stage of being in phase, entrainment is no longer needed to stay synchronized with the musical beat. That's why strategies that directly phase-lock the exerciser's movement with the music are preferred: they allow one to accurately predict the beat from the start. In contrast, strategies that require an exerciser to find the beat by him- or her-self are more demanding since they are more prone to erroneous beat prediction: phase-correction adjustments may require some effort and possibly take time to be accurate.

In addition to monitoring, the D-Jogger system can also be used to modify movement. Recently, an experiment was conducted to investigate whether runners could be sped up or slowed down by spontaneous auditory-motor synchronization (Van Dyck et al., 2015). Recreational runners were asked to run four laps of 200 m, a task that was repeated 11 times with a short break in between each running sequence. During each first lap, participants ran at their own preferred tempo without musical accompaniment. The average running tempo during the first lap was measured and served as a reference for the tempo-matched music—realized by the D-Jogger system—that was played during the second lap. In the last two laps, the music tempo was either increased or decreased by 3.00, 2.50, 2.00, 1.50, or 1.00 percent or was kept stable. In general, findings of this study showed that recreational runners are able to adapt their running cadence to tempo changes in music without being instructed to do so and even without being aware of this attunement. Evidence for an entrainment basin was discovered: the degree of entrainment with the tempo of the music dropped significantly as soon as tempo increases of 2.50 percent were introduced, and also when tempo decreases of 3.00 percent were introduced.

The study by Van Dyck et al. (2015) is based on the concept of deliberately introducing small errors in a runner's beat prediction, by slightly deviating the tempo of the music from the subject's running cadence. The human innate tendency to minimize beat prediction errors causes the subject to entrain the movement with the new musical tempo. Introducing beat-prediction errors was hereby managed through time stretching; compressing or stretching the time between the beats, resulting in faster or slower music, respectively. Another method for introducing beat-prediction errors is shifting the audio signal in such a way that the upcoming beat no longer coincides with the next predicted footfall. This concept is based on manipulating the phase angle between the moment of the beat and the footfall. Currently, studies are being conducted to assess the effects of these manipulations on human locomotion, running, and other types of rhythmical physical activity.

A crucial assumption underlying the use of the principle of rhythmic entrainment as attractor of rhythmic motor behavior is that people exhibit a spontaneous tendency to synchronize rhythmic movement to external rhythmical sound patterns. However, studies show that people may have weak beat perception (Leow et al., 2014), and may not always exhibit the spontaneous tendency to phase-synchronize to a musical beat (Buhmann et al., 2016).

The D-Jogger method described above relies on a two-step process; a musical rhythm becomes first automatically aligned to an individual's motor rhythm (cf. sonification) while successively, period and/or phase manipulations are applied to the musical rhythm. In this second step, the music becomes an external stimulus, and one relies on the principle of spontaneous entrainment to guide people's motor rhythms. However, it would be of interest to further study how an additional sonification of motor rhythms during tempo/phase manipulations of external music may contribute to spontaneous entrainment to this music. This additional sonification, which would stay aligned to motor rhythms, makes the discrepancy between the external musical rhythm and internal motor rhythms more evident, which in turn may reinforce motor adaptation in order to resolve this discrepancy. On top of that, a well-considered sonification design could instigate the feeling of actually participating in the musical outcome, as in a music performance. This feeling of control relates to the concept of “agency,” which refers to the subjective sense of having voluntarily control over actions and their outcome.

#### Auditory-motor association learning

Interaction with a biofeedback system can exploit auditory-motor association schemes. These schemes, or “internal models,” can be conceived as predictions of auditory consequences of actions (Maes et al., 2014). Based on a learning process, one can reliably predict the auditory outcome of performed actions, allowing the intentional production of certain sounds. This ability of prediction and control may lead to intense feelings of pleasure and reward, as in (social) music performance. Now, when a mismatch occurs between the expected and the actual auditory outcome of performed actions (i.e., a prediction error), one has the spontaneous tendency to adapt one's actions to minimize prediction errors and realize the intended outcome (cf. reward) (Lalazar and Vaadia, 2008; Krakauer and Mazzoni, 2011; Van Der Steen and Keller, 2013). In that sense, learned auditory-motor associations may function as “attractors” of coordinated behavior of humans. Based on this principle, we propose a sonification strategy that is implemented into two distinct steps. In a first step, actions become coupled to perceptual (i.e., auditory) outcomes through an associative learning process. In a second step, the auditory outcomes become altered, which is assumed to lead to spontaneous motor adaptation in a well-specified manner.

To test this idea, we conducted an experiment using a finger-tapping paradigm (Maes et al., 2015). Participants were instructed to perform on a typical synchronization-continuation task. Each finger-tap triggered a piano tone of which the amplitude's decay curve exponentially decreased in a way the tone exactly fitted the target interval (i.e., 1100 ms). Thus, the duration of the target interval was represented/sonified by this piano tone. In the synchronization phase, participants learned to associate this relationship between the interval that needed to be tapped out (action), and the auditory tone (perception). In the continuation phase, we assumed that participants could rely on the tone's decay curve to time their actions; namely that they would tap at the moment that the previous tone ceased (cf. “sensorimotor timing strategy”). Now, if people would actually deploy this sensorimotor timing strategy, this entailed that a gradual alteration of the tones' duration (shorter/longer) throughout the continuation phase, would lead to corresponding changes in interval production rate. Namely, a gradual shortening of the tones' duration would entail a speeding up, while a lengthening would entail a slowing down. The results of the experiment provided evidence for the former, and were interpreted based on error-correction and corresponding motor adaptation mechanisms. This study outlines important mechanisms underlying temporal sensorimotor coordination, which are of interest for further implementation in sonification strategies in the domain of sports and rehabilitation.

### The role of novelty and surprise

Although the ability to anticipate and control auditory events is a potent source of reward, music that is too repetitive, simple and conventional will not sustain reward responses. Current learning theories suggest that learning processes are grounded in occurring discrepancies (errors) between what is expected/predicted and what actually happens (Schultz, 1998, 2007; Waelti et al., 2001; Hazy et al., 2010). In these studies, it is shown that dopamine neurons encode reward learning prediction errors; a positive response of dopamine neurons occurs when a reward is given unexpectedly, while this response gradually decreases as that reward becomes increasingly predictable. In line with this finding, Fiorillo et al. (2003) found that dopamine response is maximal when the uncertainty of a given reward outcome is highest, and decreases when reward outcome becomes more predictable. These findings advocate the importance of tension and uncertainty in musical compositions in order for them to be experienced interesting and pleasant (Gebauer et al., 2012). In the context of musical biofeedback systems for sports and motor rehabilitation, these findings stress the necessity to include aspects of surprise and novelty into sonification designs and strategies in order to support learning, self-regulation, and motivation processes.

### The role of expression

Leman (2016) defines music as emergent patterns endowed with expression. Expressive cues in music have a relationship with sound-encoded human gestures and have a special appeal as biosocial signal that promotes the formation of an interaction pattern between partners involved (often called sender and receiver). Expression in music works as an affordance, that is, an opportunity for expressive responding. The assumption is that musical patterns with expressive cues have a higher affordance for expressive responses than musical patterns without these expressive cues. A good example is the difference between deadpan piano music (e.g., a MIDI score played on a MIDI grand piano) and the same piano music onto which expressive cues are added (Flossmann et al., 2012). Listeners tend to respond more to the expressive music than to non-expressive music. Leman et al. (2013) and Buhmann et al. (2016) provide clear evidence that musical expression (activating vs. relaxing expression) can affect the velocity of walking, with a maximum effect size of about plus or minus 10% velocity increase or decrease in task-related settings and 3% velocity increase or decrease in spontaneous settings. Whether this effect is obtained by means of arousal-mediation, or by means of cross-model audio-motor overlapping brain regions is a topic of future research. The main observation is that expression seems to function as a facilitator for establishing a human-music interaction using the principles described above.

## Conclusion

Auditory feedback of physiological processes, motor processes, and performance parameters has been proven particularly useful in the domain of sports and motor rehabilitation, for the purpose of stress regulation, motor retraining, and motor performance optimization. Also, research has demonstrated effects of (background) music on physiological, psychological, and motor aspects. However, the use of music in biofeedback systems has been much less explored. In the current article, we asserted that the use of music in biofeedback systems might have a major added value to the benefit of psychological and physical optimization of sports and motor rehabilitation tasks. Therefore, we presented the 3Mo model to describe three main functions of music that contribute to these benefits; namely Motivation, Monitoring, and Modification. The main idea was to delineate important components and principles to take into account and exploit in sonification designs. These components and principles are explicitly oriented toward music, and fundamental knowledge on human's sensorimotor interaction with music, and on the underlying physiological and psychological processes.

Although the model delineates important concepts and principles, many challenges lie ahead in order to fully realize the potential of musical biofeedback. For instance, further research is required to define the musical and acoustic parameters giving music motivational or relaxing qualities. Also, new methods need to be developed to (automatically) analyze multilayered periodicity patterns in music. In addition, there is a need to test and further develop strategies for modification of physical movement behavior. These strategies should incorporate temporal aspects, as well as spatial and spatiotemporal aspects. Another important challenge is the further integration of physiological and physical processes. Finally, progress needs to be made in the domain of hardware and software in order to provide reliable signals to be sonified. The 3Mo model indicates that the use of sonification in the field of sports and motor rehabilitation requires close collaboration with other fields of research, including musicology, psychology, neuroscience, physiology, engineering, and art. It is only through actual collaborative research that the full potential of musical biofeedback will be discovered and put into practice.

## Author contributions

PM: Outlined the general structure of the article, and wrote the main part of the article. JB: Contributed substantially to the writing of the article with a focus on the description of empirical studies. ML: Contributed substantially with the development and shaping of the conceptual framework outlined in the article.

## Funding

This research was conducted in the framework of the EmcoMetecca II project, granted by Ghent University (Methusalem-BOF council) to ML.

### Conflict of interest statement

The authors declare that the research was conducted in the absence of any commercial or financial relationships that could be construed as a potential conflict of interest.
